# Using the GAPSED+ Equity Organising Framework to drive health equity for women and girls

**Published:** 2025-12-15

**Authors:** Lisa Johnson, Minh Ha, Wang Jing, Lana Woolf

**Affiliations:** 1 Global Equity and Inclusion Lead: The Fred Hollows Foundation, Sydney, Australia.; 2 Program and Partnerships Manager: The Fred Hollows Foundation, Sydney, Australia.; 3 Senior Project officer: The Fred Hollows Foundation, Sydney, Australia.; 4 Independent Senior Consultant: Community Powered Responses, Melbourne, Australia.

**Identifying who is being left behind is the first step towards equity.**

**Figure:**
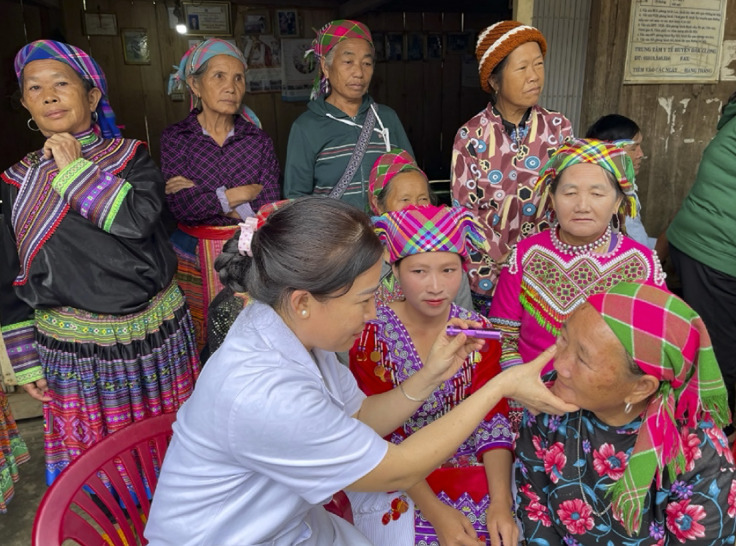
Mobile teams provided vision screening and same-day referrals in remote areas. VIET NAM

© TRANG NGUYEN, THE FRED HOLLOWS FOUNDATION CC BY-NC-SA 4.0

Too often, the very people who most need eye health services are invisible in the data, meaning we don't know if they are accessing care, or to what extent. Unless we shine a light on who is missing, why they are excluded, and how to dismantle those barriers, we risk leaving them behind.

Globally, 1.1 billion people live with vision loss, 55% of whom are women or girls. To address inequities in access to eye health, The Fred Hollows Foundation developed a framework and guidance manual that applied throughout the project cycle – from situation analysis and planning to monitoring the equity of outcomes. The GAPSED+ framework helps project planners gather information on who is and isn't using services, across key factors: **G**ender/sex, **A**ge, **P**lace of residence, **S**ocio-economic status, **E**thnicity/Indigeneity/Race/Culture, **D**isability, and a context-specific ‘**Plus**’ category for local factors such as religion, statelessness, or albinism. The GAPSED+ Framework has adapted the PROGRESS acronym for the various domains of equity, as used in public health,^1,2^ to create a tool that is specific to the eye sector.

The information gathered helps identify who is most at risk of exclusion from eye health services, and why. This enables planners to design targeted strategies to address barriers, as highlighted in the *Community Eye Health Journal* issue Eyes on equity: advancing eye health for women and girls (bit.ly/424pRMH).^3^

## Piloting the GAPSED+ framework to enhance women's cataract surgery uptake in remote Viet Nam

In 2023, The Foundation implemented a project in three northern provinces of Viet Nam – Hoa Binh, Dak Nong, and Ben Tre – to address persistently low cataract surgery rates among women in remote ethnic minority communities. Using a mixed-methods situational analysis and the GAPSED+ framework, and working in collaboration with local women's unions and departments of health, the project identified four key barriers across the **G**ender/sex, **P**lace of residence, **S**ocio-economic status, and **E**thnicity domains. Cultural expectations around women's domestic roles limited their ability to travel; eye health services were unevenly distributed, hindering access in remote areas; out-of-pocket costs disproportionately affected low-income women; and language barriers restricted access to information for ethnic communities not speaking the dominant language. The programme collaborated with partners and community-based organisations to co-design disaggregated data tools, validate findings through participatory workshops, and identify priority sites using prevalence data. Five equity-driven strategies followed:

1. **Gender-responsive targeting.** Surgical targets were informed by local baseline data, with each province aiming for 74% female participation, reflecting Rapid Assessment of Avoidable Blindness (RAAB) survey findings that 74% of people needing cataract surgery nationally are women.
2. **Community engagement.** Women's unions and ethnic minority networks co-delivered eye health education community events (e.g., local festivals, World Sight Day, International Women's Day), tailoring messages to women's needs.
3. **Mobile outreach services.** In partnership with district hospitals, mobile teams provided screening and same-day referrals in remote areas, reducing mobility-related barriers for women.
4. **Culturally appropriate information, education, and communication (IEC) materials.** Materials were translated into local languages, incorporating locally resonant visuals and gender equity messaging, and pre-tested with women's groups for relevance and clarity.
5. **Transport subsidies.** Means-tested vouchers covered round-trip transport for older women, women from ethnic minorities, and women with disabilities, validated through participatory criteria developed with local leaders.

While the early results are promising, it is still too soon to fully assess long-term impact. The priority is to continue monitoring and analysis to identify effective strategies and needed adjustments. Building on these insights, the Viet Nam programme is developing a new initiative to strengthen women's leadership in eye health, ensuring that women's voices and experiences drive sustainable, equitable change.

The full position statement: “A framework for addressing equity in access to eye care” is available at bit.ly/3JZzqah. The GAPSED+ Guidance Manual is available bit.ly/4p7p86Z.

## References

[1] O’Neill J, Tabish H, Welch V (2014). Applying an equity lens to interventions: using PROGRESS ensures consideration of socially stratifying factors to illuminate inequities in health.. J Clin Epidemiol..

[2] Ramke J. (2016). Measuring inequality in eye care: the first step towards change.. Community Eye Health..

[3] (2025). Community Eye Health Journal.

